# Bipolar Disorder and Borderline Personality Disorder: A Diagnostic Challenge

**DOI:** 10.1192/j.eurpsy.2023.1467

**Published:** 2023-07-19

**Authors:** L. M. Lopes, M. Matias, M. Marques, I. M. Lopes, J. Reis

**Affiliations:** 1 Psychiatry, Unidade Local de Saúde do Alto Minho, EPE, Viana do Castelo; 2 Psychiatry, Centro Hospitalar Barreiro Montijo EPE, Barreiro, Portugal

## Abstract

**Introduction:**

Bipolar disorder (BD) and borderline personality disorder (BPD) appear as prevalent psychiatric conditions, with both being associated with increased morbidity and elevated suicide rates.

Borderline personality disorder is characterized by emotional instability and impulsivity, and patients are susceptible to dangerous behaviors such as unsafe sexual behavior, eating disorders and substance abuse. On the other hand, mania in BD patients contributes to impulsivity, hypersexuality and poor judgment, as well as a lack of inhibition. In both cases, individuals are at a higher risk of committing suicide due to emotional dysregulation and impulsive behaviours.

The symptomatic overlap in BPD and BD is prone to discussion and may lead to misdiagnosis. Some studies suggest that BPD and BD may lie on a spectrum, while others advocate them as separate entities that may coexist.

**Objectives:**

To explore the clinical challenges associated with both the differential diagnosis and the comorbidity of BD and BPD.

**Methods:**

We performed a non-systematic review of the literature using the database PubMed and the following keywords: *bipolar disorder borderline personality disorder comorbidity*

**Results:**

Patients with borderline personality disorder seem to have significantly greater odds of a previous bipolar misdiagnosis, but no specific borderline criterion was shown unique in predicting this outcome.

Nonetheless, research studies have reported that approximately 20% of BPD were also diagnosed with BD and, similarly, 10% of people with BD I and 20% with BD II were diagnosed with BPD.

Comorbid BPD and BD are associated with marked psychosocial disability, with patients being more impulsive and aggressive than those with BPD and BD alone. They are also highly susceptible to anxiety disorders like obsessive-compulsive disorder (OCD), post-traumatic stress disorder (PTSD), and somatoform disorders. Studies have shown that comorbid BPD has a negative impact on the clinical course of Bipolar Disorder, as the patients have an unfavorable illness trajectory, with earlier onset of mood symptoms,higher likelihood of hospitalization, longer treatment duration and worse response to treatment. They are also at higher risk for depression and persistent unemployment, increased risk drug abuse, higher suicide risk, and greater utilization of ECT, compared to patients with BPD or BD alone.

**Conclusions:**

A careful review of the patient’s history must be done to properly distinguish between BPD and BD and to make the right diagnosis. Further studies are needed to clarify the risk factors associated with the comorbidity between these two disorders, in order to develop effective clinical strategies for optimal outcomes for patients with both disorders, improve their clinical course and prevent the increased risk of suicidality.

**Disclosure of Interest:**

None Declared

